# An exploratory spatial analysis of geographical inequalities of birth intervals among young women in the Democratic Republic of Congo (DRC): a cross-sectional study

**DOI:** 10.1186/1471-2393-14-271

**Published:** 2014-08-13

**Authors:** Tobias F Chirwa, Jocelyn N Mantempa, Felly Lukumu Kinziunga, Joseph D Kandala, Ngianga-Bakwin Kandala

**Affiliations:** Division of Epidemiology and Biostatistics, School of Public Health, University of the Witwatersrand, Johannesburg, South Africa; Department of Population and Development studies, University of Kinshasa, Kinshasa, Democratic Republic of Congo; Direction d’inventaires et Aménagement Forestières (DIAF), Ministère de l’Environnement, Conservation de la Nature et Tourisme, DRC, Kinshasa, Democratic Republic of Congo; Warwick Medical School, Division of Health Sciences; Populations, Evidence and Technologies Group, Warwick Evidence, University of Warwick, CV4 7AL Coventry, UK; Malaria Public Health and Epidemiology Group, Centre for Geographic Medicine, University of Oxford, KEMRI-University of Oxford-Wellcome Trust Collaborative Programme, Nairobi, Kenya

## Abstract

**Background:**

The length of time between two successive live births (birth interval), is associated with child survival in the developing world. Short birth intervals (<24 months) contribute to infant and child mortality risks. Contraceptive use contributes to a reduction in short birth intervals, but evidence is lacking in the DRC. We aimed to investigate the proportion of short birth intervals at the provincial level among young women in the DRC.

**Methods:**

Data from the Demographic and Health Survey undertaken in the DRC in 2007 were analyzed. Logistic regression and Bayesian geo-additive models were used to explain provincial inequalities in short birth intervals among women of reproductive age and young women. Posterior odds ratio (OR) and 95% credible region (CR) were estimated via Markov chain Monte Carlo (MCMC) techniques. Posterior spatial effects and the associated posterior probability maps were produced at the provincial-level to highlight provinces with a significant higher risk of short birth interval.

**Results:**

The overall proportion of short birth intervals among all women of reproductive age (15–49 years) and young women (15–24 years) were 30.2% and 38.7% respectively. In multivariate Bayesian geo-additive regression analyses, among the whole sample of women, living in rural areas [OR = 1.07, 95% CR: (0.97, 1.17)], exclusive breastfeeding [1.08 (1.00, 1.17)] and women with primary education [1.06 (1.00, 1.16)], were consistently associated with a higher risk of short birth intervals. For the young women, none of the factors considered were associated with the risk of short birth interval except a marginal effect from the lack of education. There was a spatial variation in the proportion of women reporting short birth intervals and among all women of reproductive age across provinces, with Nord-Kivu [1.12 (1.02, 1.24)], Sud Kivu [1.17 (1.05, 1.29)] and Kasai Occidental [1.18 (1.06, 1.32)] reporting a higher risk of short birth intervals. For young women, the higher risk provinces were Nord-Kivu [1.22 (1.00, 1.54)] and Sud Kivu [1.34 (1.14, 1.63)].

**Conclusions:**

This study suggests distinct geographic patterns in the proportion of short birth intervals among Congolese women, as well as the potential role of demographic and geographic location factors driving the ongoing higher youth fertility, higher childhood and maternal mortality in the DRC.

## Background

A birth interval is the length of time between two successive live births [1–4]. Longer time periods between births allow the next pregnancy and birth to be at full gestation and growth [2]. It has an influence on mother and child health. Several fertility analyses attested that short birth intervals (<24 months) had a negative impact on the health and nutritional status of children and increased their risk of dying [2–4]. Births too close together are associated with schizophrenia in offspring [5] and hinder the physiological ability of mothers and, thus, expose them to complications during and after pregnancy [2–4].

Women in developing countries have shorter birth intervals than they would personally prefer. The main reason for short birth intervals is that many women in developing countries do not use contraception after birth and therefore are likely to become pregnant once fecundity returns [6]. Contraceptive use is an effective way of controlling fertility and improving maternal and child health through birth spacing [2, 5]. Through modelling, it has been established that current levels of contraceptive use will prevent 218 million unintended pregnancies in low-income countries during 2012, and will avert 138 million abortions (of which 40 million are unsafe), 25 million miscarriages and 118 000 maternal deaths all over the world [7]. However, the number of women who have an unmet need for modern contraception in 2012 is estimated at a staggering 222 million globally [7]. Considering that contraceptive use is low, there is a need for integrated programmes that include improved knowledge on birth intervals and its associated factors.

### Rationale and justification for the study

In 2008, an analysis of data on 844 837 women from 52 Demographic Health Survey data (DHS) from 52 different countries, showed that 54.3% of the recent two births in developing countries were birth intervals of less than 24 months, with children born in those intervals 2.27 times more likely to die before their fifth birthday compared to children born in intervals longer than 24 months [2]. Since then, the proportion of short birth intervals has reduced, although the levels are still worrying. Latest DHS results published in 2012 still show high levels of short birth intervals in many African countries (Uganda: 25.3%; Ethiopia: 20.4%; Rwanda: 20.0% and Cameroon: 21.3%) [8]. However, for the same countries, for example in Uganda, 34.3% of women aged 15–49 years want to delay the time to have a child or more children but are not using any method of contraception. However, we acknowledge that this group could be different from the group on which the reported 25.3% short birth intervals were based. There is an average of one in four African women with unmet needs for Family Planning (FP).

The Democratic Republic of Congo (DRC) is not an exception and follows the same pattern. The last 2007 DHS results have revealed that births which have short intervals have led to high maternal and child deaths (549 women per 100,000 live births and 97 children per 1000 live births). Under-five mortality rates (U5MRs) was associated with birth interval. U5MRs were higher (214.3 death per 1000 live birth among children from short birth intervals compared with 133.5 death per 1000 live birth among those from long birth intervals. These results remained statistically significant after multiple adjustment (OR & 95% CI: 1.14(1.04, 1.26) [9]. Twenty-six percent of births occurred in an interval of less than 24 months and 9.4% of children had a low birth weight. With an average of 5.8 children per woman at the end of her reproductive period, the DRC has one of the highest fertility rates in Sub-Saharan Africa (SSA). About 11% of women have more than 4 children, some of which were unplanned. In addition, 19.5% want longer birth intervals and 5% want to limit the number of births but they do not have access to contraception.

Also of importance is the provincial inequality between the use of modern contraception and short birth intervals which requires that the spatial dimension be fully investigated. Such unique analysis is often not included in conventional statistical analysis approaches. Many developing countries have substantial geographic variations in birth intervals although the factors shaping these variations are little understood [10]. Although geographical variations in demographic, cultural and socio-economic factors exist in the DRC, it does not have birth interval maps to show the varied distribution and its potentially associated factors.

A fundamental factor in the study of contraceptive decision-making is the space/environment in which people operate [11]. It is now an increasingly recognized problem that to achieve the MDG goals in developing countries, one must integrate the spatial dimension [10, 12–15]. This proposed study involving spatial analysis sheds light for policymakers on repositioning family planning methods, specifically an understanding of birth intervals.

The purpose of this article is to identify birth spacing inequalities in provinces in the DRC and check the possible association between the use of contraception and birth intervals among Congolese women in general and young women in particular.

## Methods

### Study population

The study population is women of reproductive age (15–49 years old) in the DRC. The study is based on the Demographic Health Surveys (DHS), which are periodic cross sectional health surveys funded by USAID (the U.S. Agency for International Development’s) Bureau for Global Health. The DHS includes a number of modules on demographics and household affluence; fertility, reproductive health, maternal and child health; nutrition, knowledge and practice related to HIV/AIDS (DHS, 1990–2004). Data from the DHS undertaken in the DRC in 2007 were used. The objectives, organisation, sample design and questionnaires used in the DHS surveys are described elsewhere (14).

### Socio-spatial context of the study population in the DRC

The DRC has an estimated population of nearly 70 million, which is sparsely populated in relation to its area (2, 344858 Square Km), with as many as 250 ethnic groups living in the DRC, of which the majority are Bantu. Together, Mongo (from the Equateur province), Luba (from the 2 Kasai and Katanga provinces) and Kongo (from Bas-Congo and Bandundu provinces) peoples (Bantu) and Mangbetu-Azande peoples constitute around 45% of the population distributed across the 11 provinces including the capital city Kinshasa with a mix of all ethnic groups and about 600,000 Pygmies, the aboriginal people of the DR Congo living in the Equateur province.

For this study, the spatial distribution of the survey participants across the 11 provinces of the DRC is shown in Table 1. In brief, this was a representative random probability sample of households designed to provide estimates of health, nutrition, education and children’s indicators at the national level, for urban and rural areas, and for the 11 provinces. The sample selection strategy is described elsewhere. Standard Enumeration Areas (SEAs) were selected, with at least one cluster in each province. The sample districts were selected following the Expanded Programme on Immunisation (EPI) Cluster Sampling Technique. Within each cluster, the required number of villages was selected through the application of the EPI sampling technique. Within each village the required number of households was selected randomly by spinning a bottle. Full technical details of the sample are included in (14).

**Table 1:** **Unadjusted and fully adjusted Odd ratios (OR) and 95% Confidence Interval (CI) of short birth interval (<=24 months) for all women of reproductive age (15–49 years) based on logistic regression and Bayesian geo-additive model**

Variables	n = 7172%	Un-Adjusted		Adjusted		Bayesian	
		Odds ratio	95% C.I.	Odds ratio	95% C.I.	Odds ratio	95% C.I.
**Province**							
Kinshasa	23.5	RC		RC		0.85	0.74, 0.95
Bas-Congo	24.2	1.036	0.79, 1.36	0.99	0.75, 1.32	0.85	0.77, 0.94
Bandundu	23.4	0.994	0.77, 1.28	0.97	0.74, 1.27	0.88	0.77, 0.96
Equateur	31.4	1.485	1.17, 1.88	1.39	1.09,1.80	1.06	0.97, 1.17
Orientale	26.7	1.181	0.91, 1.54	1.09	0.82, 1.45	0.94	0.84, 1.06
Nord-Kivu	35.3	1.770	1.39, 2.26	1.62	1.25,2.11	1.12	1.02, 1.24
Maniema	27.6	1.241	0.97, 1.59	1.13	0.87, 1.46	0.94	0.83, 1.05
Sud-Kivu	38.0	1.993	1.57, 2.53	1.83	1.42, 2.37	1.17	1.05, 1.29
Katanga	30.8	1.446	1.14, 1.84	1.37	1.07,1.76	1.02	0.93, 1.11
Kasaï Occident	31.1	1.470	1.16 1.87	1.37	1.06,1.77	1.18	1.06, 1.32
Kasaï Oriental	37.0	1.909	1.52, 2.40	1.78	1.39, 2.27	1.04	0.93, 1.15
**Location of residence**							
Urban	28.9	RC		RC			
Rural	31.1	1.109	1.00, 1.23	1.00	0.89, 1.14	1.07	0.97, 1.17
**Household economic status**							
low income household	30.5	0.992	0.88, 1.11			0.98	0.80, 1.01
middle income household	28.6	0.902	0.78, 1.04			0.86	0.77, 0.94
Higher income Household	30.7	RC					
**Education**							
No education	32.1	1.227	1.07, 1.41	1.07	0.91, 1.25	1.08	0.97, 1.19
Primary	30.8	1.157	1.03, 1.30	1.06	093, 1.22	1.06	1.00, 1.16
Secondary and higher	27.8	RC		RC			
**Age**							
15-19	47.3	2.584	1.61,4.14	2.513	1.56, 4.04		
20-24	37.9	1.754	1.25, 2.47	1.790	1.27, 2.52		
25-29	30.1	1.238	0.88, 1.73	1.309	0.93, 1.84		
30-34	29.2	1.187	0.85 1.66	1.253	0.89, 1.76		
35-39	25.9	1.005	0.71, 1.42	1.054	0.74, 1.50		
40-44	24.7	0.945	0.66, 1.36	.994	0.69, 1.44		
45-49	25.8	RC		RC			
**Use of contraception**							
Not using	31.0	1.125	0.90, 1.41			0.97	0.85, 1.08
Traditional & folkloric	27.4	0.945	0.74, 1.21				
Modern method	28.5	RC					
**Breastfeeding practice**							
Never	30.0	1.021	0.91, 1.14			1.07	0.99, 1.15
Exclusive	32.4	1.144	0.99, 1.31			1.08	1.00, 1.17
Mixed	29.6	RC					

RC: Reference Category.

The nationally representative sample from the 2007 DHS-DRC were collected from 8,886 households, and complete interviews were conducted with 9,995 women aged 15–49 and 4,757 men aged 15–59 with a response rate of 95%.

There were few participants with missing data on contraception choice, proceeding birth intervals and other covariates. Thus, the current analysis is based on 7172 women of reproductive age (15–49 years) with a complete set of data.

### Outcome measurement

The outcome variable for this study is short birth interval defined as women reporting a preceding birth interval of  ≤ 24 months (short birth interval) and coded yes or no. We choose a binary outcome, because one can estimate the likelihood of short birth interval in a given province, while accounting for a number of potential covariates.

### Covariates

**Figure 1:**
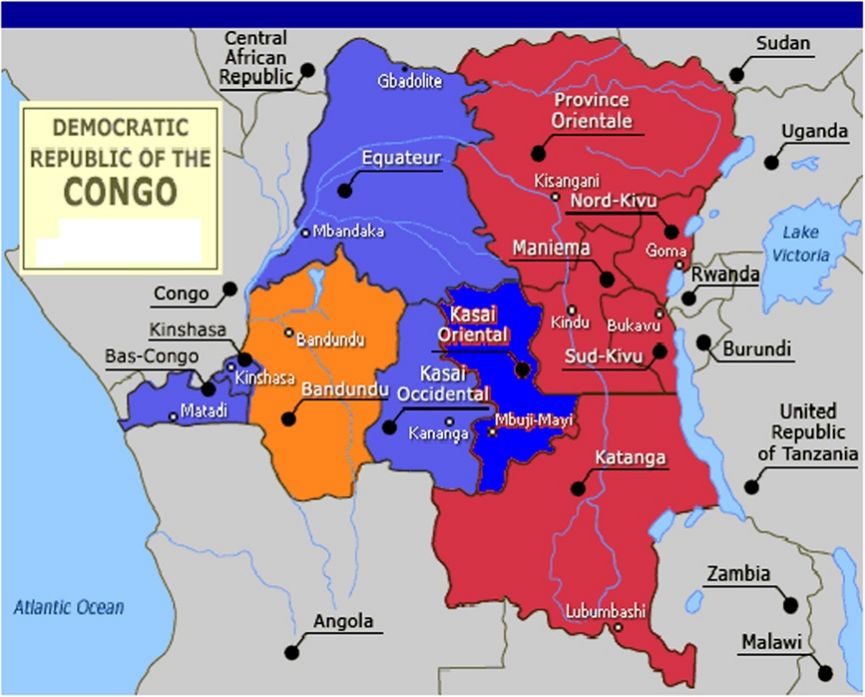
**Map of the Democratic Republic of Congo showing 11 provinces.** Provinces highlighted in red colour are conflict-affected areas.

The main exposure variable investigated was the respondents’ geographic location, i.e. administrative province of residence (Figure 1), in addition to various individual-level and household-level control variables such as socio-demographics and household socio-economic status, as explained below, or breastfeeding known to be associated with contraception choice or short birth intervals. The respondent’s age at the time of survey was also included as an indicator of the birth cohort of the participant. Other socio-demographic covariates were education of the respondent (no education vs. primary, secondary and higher education), household socio-economic status based on level of income (low and middle income versus high income households), breastfeeding (never and exclusive versus mixed feeding). Finally, environmental factors included place (locality) of residence (rural vs. urban) and province of residence of the respondents.

### Statistical analysis

We sub-divided the analysis into two groups: the first analysis used data on all women of reproductive age (15 to 49 years old: n = 7172) and the second analysis was focused on young women defined as women of reproductive age between 15 to 24 years old (n = 1389).

The outcome variable, short birth interval was cross-tabulated by the 11 provinces of the DRC, and by reported current use of any modern reversible contraception, depotmedroxyprogesterone acetate (DMPA) and intrauterine device. Further associations between short birth intervals and demographic, environmental and behavioural variables were explored. The statistical significance of these apparent associations between potential risk factors and the proportion of women reporting a proceeding birth interval of ≤ 24 months and use of contraception was explored using chi-square (χ^2^) and Mann–Whitney *U*-tests, as appropriate.

Univariate logistic and bayesian regression models (detailed below) were also fitted to investigate factors significantly associated with outcome variable on their own. Further, multiple regression models were fitted for factors found to be significant at the univariate level analysis. To capture the impact of the women’s physical environment on the use of modern contraception, the province of residence of the women during the interview period, was modelled as spatial effects only in the Bayesian framework. In all the analysis, significance was considered at 5% level.

In the analysis of household survey data, commonly adopted models are standard logistic regression models, which use linear index functions. However, they do not allow adjustment for spatial effects or correlation between clusters. For example, the DHS uses cluster-sampling to draw upon women respondents via multistage sampling of enumeration areas. The levels include villages/communities; a sample of households within the selected communities; and finally, all women respondents (aged 15–49 years) in the sample households. Our proposed spatial analysis methodologies can be referred to as spatial epidemiological analysis, which are increasingly being used to describe studies that assess spatial dependence and proximity influencing health outcomes in a flexible way different to traditional analysis of incorporating region of residence as an explanatory variable.

Thus, to account for spatial autocorrelation and possible nonlinear effects of continuous risk factors in the proportion of women reporting a preceding birth interval of ≤ 24 months with the proportion reporting current use of any modern reversible contraception at the provincial level in the DRC, we applied a unified approach by using a geo-additive semi-parametric mixed model. The model employed a fully Bayesian approach using Markov Chain Monte Carlo (MCMC) techniques for inference and model checking (17–18). The response variable was defined as *y*_*i*_ *= 1 if women i reported short birth intervals, otherwise y*_*i*_ *= 0.* The standard measure of effect was the odds ratio (OR) compared to the posterior odds ratio (POR) and 95% credible region (CR) as reported in this method. This bayesian analysis was carried out using version 2.0.1 of the BayesX software package, which permits Bayesian inference based on Markov chain Monte Carlo (MCMC) simulation techniques (20).

Adjusted (marginal) ORs of short birth intervals across provinces were obtained from standard logistic regression models, with Kinshasa used as the reference category because of the lowest observed proportion of women with short birth intervals (see Table 1). The adjustment was done for factors that were univariately associated with outcome. Multiple Bayesian geo-additive regression models were used to evaluate the significance of the posterior OR determined for the fixed, non-linear effects and spatial effects.

## Results

The overall proportion of short birth interval among all women of reproductive age (15–49 years old) and young women (15–24 years old) were 30.2% and 38.7% respectively. Table 1 shows results from both standard logistic regression models (unadjusted and adjusted) and the geo-additive semi-parametric mixed model for all women of reproductive age. The results show that there was a statistical significant association between short birth intervals and province of residence (p < 0.001), location of residence (p < 0.05), age (p < 0.001), level of education (p < 0.001) and use of contraception (p < 0.005).

Kinshasa, the capital city of DRC, and her neighbouring provinces of Bandundu and Bas-Congo had less proportion of women with short birth intervals in comparison to other provinces. They have respectively, 23%, 24% and 23% of women with short birth intervals, which are below the national average. Sud-Kivu had the highest proportion of short intervals (38.0%), one percentage more than Kasai-oriental (37%). In general, the women of rural areas reported a higher proportion of short birth intervals (31.1%) compared to those of urban areas (28.9%).

There is an interesting association between education and short birth intervals. The proportion of women with short birth intervals decreases from 32.1% in those with no education to 27.8% in those with higher education.

The results of Table 1 show the association between short birth intervals among women, province of residence and age. Sud-Kivu, the province of higher prevalence of short birth intervals (38.0%), has the highest odds ratio (OR 1.83, 95% CI 1.42 to 2.37) in the adjusted analysis in reference to women of Kinshasa. Those who were less than 25 years of age were more likely to have short birth intervals. Young women of 15–19 years (OR 2.51, 95% CI 1.56 to 4.04) were nearly 2.5 times more at risk of short birth intervals than those aged 45–49 years old.

Table 2 shows results from both standard logistic regression models (unadjusted and adjusted) and the geo-additive semi-parametric mixed model for only young women of reproductive age. For this group, short birth intervals were associated with the province (p < 0.001), household economic status (p < 0.001) and age (p < 0.05). However, education was no longer associated with short birth intervals. Young women of Sud-Kivu got the highest proportion of short birth intervals (53.9%) compared to those in other provinces. About 47% of young women who are less than 20 years of age had birth intervals of more than 24 months compared to 37.9% in those who were 20 years and more.

**Table 2:** **Unadjusted and fully adjusted Odd ratios (OR) and 95% Confidence Interval (CI) of short birth interval (<=24 months) for young women of reproductive age (15–24 years old) based on logistic regression and Bayesian geo-additive model**

Variables	n = 1389%	Un-Adjusted		Adjusted		Bayesian	
		Odds ratio	95% C.I.	Odds ratio	95% C.I.	Odds ratio	95% C.I.
**Province of residence**							
Kinshasa	35.7	RC		RC		0.85	0.69, 1.03
Bas Congo	26.9	0.66	0.34, 1.30	0.89	0.44, 1.77	0.80	0.64, 0.97
Bandundu	22.7	0.53	0.27, 1.03	0.73	0.36, 1.47	0.80	0.62, 1.02
Equateur	33.1	0.89	0.51, 1.56	1.22	0.67, 2.24	0.98	0.91, 1.15
Orientale	30.4	0.79	0.43, 1.45	0.98	0.52, 1.86	0.91	0.77, 1.08
Nord-Kivu	47.9	1.65	0.95, 2.88	2.07	1.16, 3.69	1.22	1.00, 1.54
Maniema	35.7	1.00	0.58, 1.73	1.32	0.74, 2.35	1.02	0.88, 1.20
Sud-Kivu	53.9	2.11	1.22, 3.65	2.58	1.46, 4.56	1.34	1.14, 1.63
Katanga	43.7	1.40	0.79, 2.48	1.71	0.94, 3.12	1.15	0.92, 1.42
Kasaï Occidental	38.7	1.14	0.65, 1.99	1.39	0.78, 2.49	1.07	0.91, 1.29
Kasaï Oriental	42.2	1.31	0.77, 2.25	1.59	0.91, 2.77	1.03	0.87, 1.22
**Location of residence**							
Urban	41.4	RC					
Rural	37.3	0.84	0.67, 1.06			1.03	0.86, 1.21
**Household economic status**							
Low income households	37.6	0.73	0.57, 0.93	0.75	0.57, 0.97	0.78	0.65, 0.96
Middle income households	30.6	0.53	0.39, 0.73	0.55	0.39, 0.77	0.63	0.82, 1.00
High income households	45.3	RC		RC			
**Education**							
No education	38.9	1.08	0.80, 1.44			1.07	0.86, 1.31
Primary	39.5	1.11	0.85, 1.45			1.12	0.91, 1.35
Secondary and higher	37.1	RC					
**Age**							
15-19	47.3	1.47	1.02,2.12	1.36	0.93, 1.97		
20-24	37.9	RC		RC			
**Use of contraception**							
Not using	39.2	1.21	0.87, 1.70			0.99	0.73, 1.32
Traditional or folkloric	34.7	1.31	0.76, 2.24				
Modern method	41.0	RC					
**Breastfeeding practice in DRC**							
Never	38.6	0.96	0.74, 1.25			1.03	0.82, 1.24
Exclusive	37.0	0.90	0.68, 1.19			0.91	0.77, 1.07
Mixed	39.5	RC					

Similar results as those for all women are observed by province. Young women (Table 2) of Sud-Kivu were among the ones who had reported the highest proportion of short birth intervals. They were nearly 3 times more likely to have short birth intervals than young women in Kinshasa (OR 2.58, 95% CI: 1.46 to 4.56). It also appears that young women of low income and middle income households were less likely (OR 0.75, 95% CI: 0.57 to 0.97) and (OR 0.55, 95% CI: 0.39 to 0.77) to have short birth intervals than their counterparts of higher income households.

Tables 1 and 2 also display posterior odds ratios of short birth intervals from the multiple bayesian geo-additive models. Results from both standard logistic regression and multivariate Bayesian geo-additive analyses (right-hand column) support the role of women education and the place of residence as risk factors for short birth intervals for all women but not for younger women. Specifically, for all women of reproductive age with primary education (Table 1) the posterior odds ratio (OR) and 95% credible region (CR) was 1.06 (1.00, 1.16).

**Figure 2:**
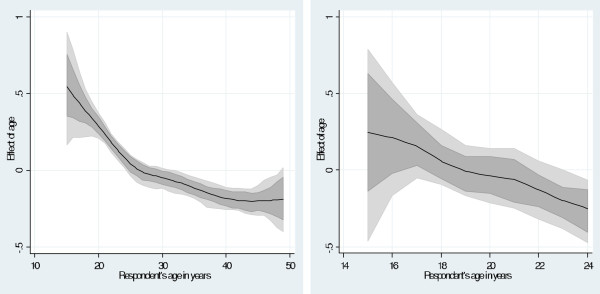
**Estimated nonparametric effect of respondent’s age on short birth interval for (a) all women (15–49 years old) and (b) young women (15–24 years old), shown with posterior means and their 80% credible regions.**

For younger women (Table 2), those in low income households [0.78 (0.65, 0.96)] were associated with lower odds of short birth intervals compared to those in high income households. Furthermore, there was a clear inverse negative nonlinear association between respondent’s age and the risk of short birth intervals. As expected, for younger women the likelihood of short birth intervals decreased with age. For all women, the likelihood of short birth intervals also decreased as age of the respondent increased, after peaking at 15 to 19 years as shown in Figure 2 using a flexible nonlinear curve.

**Figure 3:**
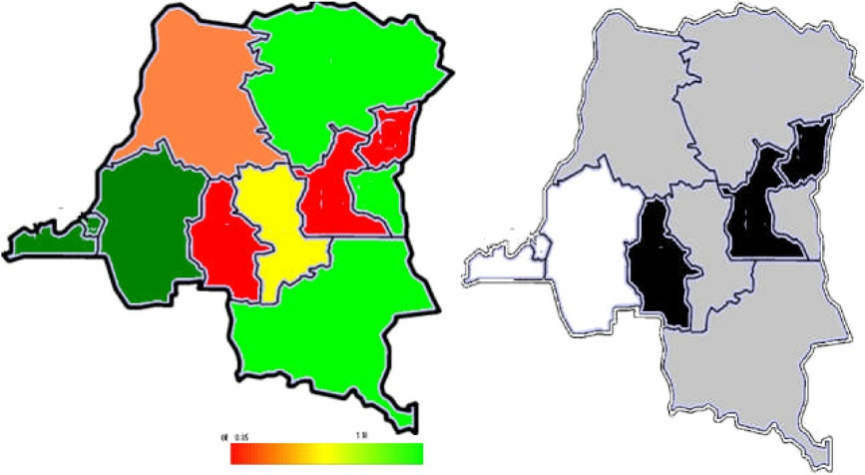
**Total residual spatial effects at province level in DRC, of short birth interval showing the posterior odds ratio and corresponding posterior probabilities at 80% nominal level.**

**Figure 4:**
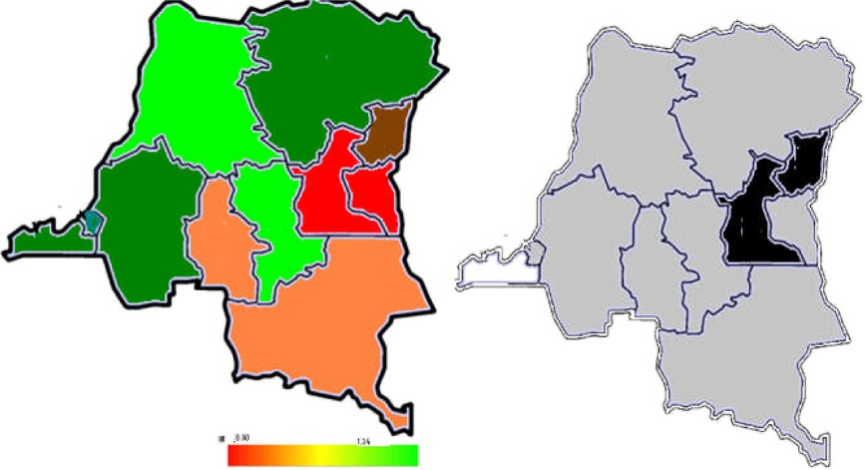
**Total residual spatial effects at province level in DRC, of short birth interval among the youth showing posterior odds ratio and corresponding posterior probabilities at 80% nominal level.**

Spatial analyses showed a striking variation in short birth intervals among all women of reproductive age across provinces, the highest likelihood of short birth interval is in Kasai Occident [1.18 (1.06, 1.32)], Sud Kivu [1.17 (1.05, 1.29)], the lowest in Kinshasa [0.85 (0.74, 0.95)], Bas-Congo [0.85 (0.77, 0.94)] and Bandundu [0.88 (0.77, 0.96)]. For the young women, Sud-Kivu [1.34 (1.14, 1.63)] and Nord Kivu [1.22 (1.00, 1.54)] had the highest likelihood of short birth intervals. All other provinces had lower birth intervals, especially Bas-Congo and Bandundu (Tables 1 and 2 and Figures 3 and 4).

In Figures 3 and 4, the left-hand maps show estimated posterior total residual provincial odds of short birth intervals (i.e. odds after multiple adjustment for the geographical location, taking into account the auto-correlation structure in the data, the uncertainty at the province level and all subject-level traditional risk factors) in each province, with the red colour indicating the maximum posterior odds recorded while green denotes a lower odds. The right-hand maps show the 95% posterior probability maps of short birth intervals, which indicate the statistical significance associated with the total excess risk. White colour indicates a negative spatial effect (associated with a reduced risk of short birth intervals), black colour a positive effect (an increased risk) and grey colour a non-significant effect. In all, there was a consistently higher likelihood of short birth intervals in Kasai Occidental, Nord Kivu and Sud-Kivu and a lower prevalence in Kinshasa, Bas Congo and Bandundu. The covariate-adjusted provincial short birth interval differentials captured by the global provincial effects (left) besides posterior significance maps (right) of Figures 3 and 4 show a clear East-south gradient; specifically, Kasai Occidental, Sud-Kivu and Nord Kivu were significantly associated with a higher likelihood of short birth intervals, while Kinshasa, Bas Congo and Bandundu provinces were associated with a lower risk of short birth intervals. These spatial patterns confirm the observed marginal model findings shown in Table 1. For young women, Nord-Kivu and Maniema were associated with a higher likelihood of short birth intervals, while Bas Congo was associated with a lower risk of short birth interval.

## Discussion

The aim of this study was to identify birth interval spacing inequalities at the province-level in the DRC. We utilised the 2007 DRC DHS data to model and provided provincial risk factors associated with short birth intervals and also provided spatial patterns of short birth intervals among Congolese women of reproductive age by comparing these patterns with their younger counterparts within the sample.

The study is not without limitations. Since our analysis required information on preceding birth intervals of the second children born to the women sampled in the survey excluding the first birth, the analysis was conditioned on women who had at least one child leading to the problem of non-randomness of the selected sample of women. Further, although cluster sampling is a cost-saving measure, without the need to list all the households, statistically, it creates analytical problems in that observational units are not independent. Thus, statistical analyses that rely upon the assumption of independence of households drawn will be no longer valid.

However, notwithstanding those limitations, the study shows that more than 30% of births occur within a spacing of < = 24 months. The study does not show an association between the use of modern contraception and birth spacing. Similar results were found in Malawi, Tanzania and Zambia where, further on, the increased use of modern contraceptive methods was not accompanied by a decrease in percentage of short intervals between births [15]. In Ethiopia, for example, among women who used contraception, 48% reported short birth intervals [16]. Moreover, Kinshasa, where the proportion of modern contraception use is the highest, does not record the highest score in terms of birth interval spacing. Kinshasa has the same level of the proportion of short birth intervals as those observed in provinces such as Bandundu and Bas-Congo which make up the majority of the population.

The study shows that short birth intervals are equally the same for women in rural and urban areas. Perhaps, the effect of rural–urban residence has been captured by the spatial effects of the province of residence, which points to the importance of the spatial analysis for the DRC. The study is in contrast to the existing literature, which shows that differential access to better social services and information, education and employment opportunities in urban areas have brought about a variation in birth spacing by rural–urban residence [17, 18]. For the 51 countries surveyed by the DHS, women who live in rural areas were more likely than women in urban areas to have birth intervals shorter than 2 years [18–20]. However, sometimes better educated women compress child bearing into fewer years in order to participate in non-child bearing activities and hence have shorter birth intervals than less educated [19]. As opposed to results for all women, the young women’s short birth intervals are associated with the province of residence, household economic status and age. In fact, education is not associated with birth intervals among young mothers due to less variation in education levels among young mothers.

There were differences in birth intervals by age of the mother. Young mothers reported a higher likelihood of short birth intervals than older ones and this finding is similar to studies conducted elsewhere [16, 19, 20]. This could be due to greater fertility and early marriage among younger women and being economically disadvantaged to access modern contraceptives than older women [16]. Another possible explanation is the lack of family planning (FP) services for young women or the lack of access to FP in general in the country due the generalised collapse of the health infrastructure.

In summary, the association of short birth interval with the household socio-economic status, and education of the women is not a new finding and has been reported in literature. What is new and novel, using the spatial modelling, is the quantification of the spatial effects by highlighting provinces such as Nord Kivu as a province exposing women of reproductive age and young mothers to a higher likelihood of short birth intervals. The spatial effects of the provinces also capture the residual effects of short birth interval that cannot be explained by the measured factors in the sample. These spatial effects are in addition to the women’s individual risk factors, which is a novel approach. The residual spatial effects in the context of the DRC could represent unmeasured factors such as FP provision and facilities, province specific economic crisis of the health system, the accessibility and affordability of FP services including province specific cultural factors as well as the on-going conflict, which has added a devastating economic impact on the already collapsed health system.

Our methods are also able to show clearly the negative nonlinear relationship between a woman’s age and her likelihood of reporting a short birth interval. Finally, the spatial effects are able to disentangle the urban–rural apparent observed association with birth intervals within provinces. Clearly, in the context of a lack of national FP policies and collapsed health system, the advantage of urban areas has been dissolved shown by the captured spatial effects.

## Conclusions

In conclusion, this study suggests distinct geographic patterns in the proportion of short birth interval among Congolese women, as well as the potential role of demographic, socio-economic and geographic location factors driving the ongoing higher childhood and maternal mortality in the DRC. We found several consistent associations between socio-demographic variables and proportion of short birth interval in a nationwide sample of Congolese women from the 2007 DHS. The geographic analysis showed distinct patterns in the proportion and risk short birth interval across the country’s provinces, pointing to the potential influence of demographic, cultural, socio-economic and environmental factors, as well as to an increasing level of higher fertility rate, which are all driving the ongoing higher childhood and maternal mortality in these settings. Importantly, policy makers and public health practitioners can use this geographic information on short birth interval mapping for planning purposes, educational and family planning programs on birth spacing, but also in the decision making process for the allocation of public resources to the most affected areas of the population, especially for youths in these low-income provinces.
